# The Incidence of Postpartum Hemorrhage in Pregnant Women with Placenta Previa: A Systematic Review and Meta-Analysis

**DOI:** 10.1371/journal.pone.0170194

**Published:** 2017-01-20

**Authors:** Dazhi Fan, Qing Xia, Li Liu, Shuzhen Wu, Guo Tian, Wen Wang, Song Wu, Xiaoling Guo, Zhengping Liu

**Affiliations:** 1 Department of Obstetrics, Southern Medical University Affiliated Maternal & Child Health Hospital of Foshan, Foshan, Guangdong, China; 2 Department of Epidemiology and Biostatistics, School of Public Health, Anhui Medical University, Hefei, Anhui, China; 3 Department of Library, the First Affiliated Hospital, College of Medicine, Zhejiang University, Hangzhou, Zhejiang, China; 4 School of Integrated Traditional and Western Medicine, Anhui University of Chinese Medicine, Hefei, Anhui, China; University of North Carolina at Chapel Hill, UNITED STATES

## Abstract

**Background:**

The global burden of postpartum hemorrhage (PPH) in women with placenta previa is a major public health concern. Although there are different reports on the incidence of PPH in different countries, to date, no research has reviewed them.

**Objective:**

The aim of this study was to calculate the average point incidence of PPH in women with placenta previa.

**Methods:**

A systematic review and meta-analysis of observational studies estimating PPH in women with placenta previa was conducted through literature searches in four databases in Jul 2016. This study was totally conducted according to the MOOSE guidelines and in accordance with the Preferred Reporting Items for Systematic Reviews and Meta-Analyses standard.

**Results:**

From 1148 obtained studies, 11 included in the meta-analysis, which involved 5146 unique pregnant women with placenta previa. The overall pooled incidence of PPH was 22.3% (95% CI 15.8–28.7%). In the subgroup, the prevalence was 27.4% in placenta previas, and was 14.5% in low-lying placenta previa; the highest prevalence was estimated in Northern America (26.3%, 95%CI 11.0–41.6%), followed by the Asia (20.7%, 95%CI 12.8–28.6%), Australia (19.2%, 95% CI 17.2–21.1%) and Europe (17.8%, 95% CI, 11.5%-24.0%).

**Conclusions:**

The summary estimate of the incidence of PPH among women with placenta previa was considerable in this systematic review. The results will be crucial in prevention, treatment, and identification of PPH among pregnant women with placenta previa and will be contributed to the planning and implantation of relevant public health strategies.

## Introduction

Placenta previa (PP) is characterized by the abnormal placenta overlying the endocervical os, and it is known as one of the most feared adverse maternal and fetal-neonatal complications in obstetrics [1, 2]. All placentas overlying the os (to any degree) are termed previas and those near to but not overlying the os are termed low-lying [3]. There appears to be an association between endometrial damage and uterine scarring and subsequent placenta previa [4]. Meanwhile, the condition is frequently complicated by invasion of placental villi beyond the decidua basalis causing placenta accreta or increta [5]. Placenta increta can unexpectedly lead to catastrophic blood loss, multiple complications, and even death [6]. Thus, women with placenta previa have often increased the risk of postpartum hemorrhage (PPH).

Postpartum hemorrhage (PPH) is a leading cause of global maternal morbidity and mortality [7]. Maternal deaths due to PPH have increased in many countries [8, 9, 10]. It is accounting for about 30% of all pregnancy-related deaths in Asia and Africa [11, 12]. This rise in mortality rate has been attributed to a number of factors, including increasing age of women at birth, the increasing multiple pregnancy rate as a consequence of artificial reproductive techniques and the rising caesarean section rate [13, 14, 15]. The basic management of PPH consists of initial medical care and the use of uterotonic drugs and/or an intrauterine balloon [16]. When these initial therapies fail, second-line therapies, including interventional radiological techniques, uterine compression sutures, pelvic vessel ligation or new medical treatments, such as recombinant activated factor VII (rFVIIa), may be used before hysterectomy is considered to control bleeding avoid maternal death [17, 18]. Although little was known until recently about the effectiveness of these therapies in practice, it was [19] demonstrated that uterine compression sutures and interventional radiological techniques experienced higher success rates than rFVIIa and pelvic vessel ligation using a prospective cohort of women with PPH identified through UK Obstetric Surveillance System (UKOSS).

A reliable estimate of the incidence of PPH is important for informing efforts to prevent, treat, and identify causes of PPH among pregnant women with placenta previa and would be contributing to the planning and implantation of relevant public health strategies. Therefore, this study is designed to systematically review the relevant present studies which reported the incidence of PPH in pregnant women with placenta previa and has a pooled analysis of the prevalence in the overall population and subgroups of the participants. An attempt is also made to identify risk factors for the incidence of PPH by applying meta-regression analyses to the available data.

## Materials and Methods

This systematic review was conducted according to the MOOSE guidelines [20] and in accordance with the Preferred Reporting Items for Systematic Reviews and Meta-Analyses (PRISMA) standard [21]. Supporting information showed the protocol and checklist (S1 Protocol and S1 Checklist).

### Search strategy and selection criteria

Two independent authors (DF, LL) searched PubMed, Elsevier Science Direct, Cochrane Library and the Chinese National Knowledge Infrastructure database (CNKI) from database inception to 31 Jul 2016. Key words used were “placenta previa” OR “Low-lying placenta” OR “PP” AND “hemorrhage” OR “haemorrhage” OR “vaginal bleeding” AND “postpartum” in the title, abstract or index term fields. An example for the complete search strategy used for the PubMed search was presented in S1 Search Strategy. There were no language restrictions or time restrictions. Relevant eligible literatures were also scanned through cross-references of identification in the reference lists within both original and review articles.

We included observational studies (cross-sectional, retrospective and prospective studies) in participants that fulfilled the following criteria: (a) placenta previa was defined as a placenta that by ultrasound was partially or completely covering the internal os of the cervix [3]; and (b) PPH diagnosis according to blood loss of more than 500 ml for vaginal deliveries and more than 1000 ml for cesarean delivery by the American College of Obstetricians and Gynecologists (ACOG) [22]. For estimation of the incidence of PPH, we excluded studies with: (a) non-standardized diagnoses, (b) non-standardized definitions of PPH, (c) insufficient data for extraction of PPH rates. The studies were also excluded based on the following criteria: case reports, letters, review articles or editorials; or the full data was not accessible even after request from the primary/corresponding authors. In the case of multiple publications from the same study, only the most comprehensive paper or article with the largest sample size or the longest follow-up was considered.

### Data extraction

After initial evaluation, two reviewers (DF and SW) independently and carefully evaluated the articles and performed the data extraction according to the selection criteria. We extracted the following variables: first author, year of publication, survey years, study country, age (mean ± standard deviation or median, range), the number of cases of PPH and the total of placenta previa sample size. When discrepancies existed, discussion was performed via consultation with another reviewer (ZL) until a consensus was reached.

### Methodological quality assessment

The methodological quality of each study was independently assessed by two reviewers (DF and QX) via the Reporting of Observational Studies in Epidemiology (STROBE) guideline [23] which was used in previous meta-analysis [24], including our team [2]. The STROBE guideline, which was a checklist of 22 items, included 5 core components (sample population, sample size, participation rate, outcome assessment, and analytical methods to control for bias). Each core component has three options: low risk (score = 2), moderate risk (score = 1), and high risk (score = 0) (S1 Table). The total score which ranged from 0 to 10, represented the summary assessment of bias risk for each study. When there was a disagreement, it was solved by consensus of the whole team.

### Statistical analyses

Individual and pooled incidence as well as 95% confidence interval (95%CI) were calculated for each of all the included studies using the STATA 12.0 (Stata-Corp, College Station, TX, USA). Before performing an inverse-variance weighted, the incidence was transformed via the Freeman-Tukey double arcsine method [25]. Due to anticipated heterogeneity, a random effects meta-analysis was employed. The inverse variance methods and DerSimonian-Laird random-effects model meta-analysis was used to determine the weight of each study [26]. Statistical heterogeneity was evaluated by the chi-square test on Q statistic, which was quantified by the I-square values, assuming that I-square values 25, 50 and 75% were nominally assigned as low, moderate, and high estimates, respectively [27]. To investigate potential sources of heterogeneity, subgroup analyses and meta-regression were performed to find any possible sources using the following grouping variables: type of placenta previa, geographical region, maternal age, gestational week, data collection period, percentage of potential characteristics (prior cesarean sections, multiparous, and anterior position of placenta) and study quality. Furthermore, in the entire dataset, we conducted subgroup analyses (including χ^2^ tests, odds ratios) to investigate different types of placenta previa and geographical regions. Sensitivity analysis was performed to assess whether one or more studies influenced the overall results. Potential publication bias was tested using the funnel plot and the method of Egger’s regression and Begg’s test. P ≤ 0.05 indicated the presence of statistically significant.

## Results

### Characteristic results

**Fig 1:**
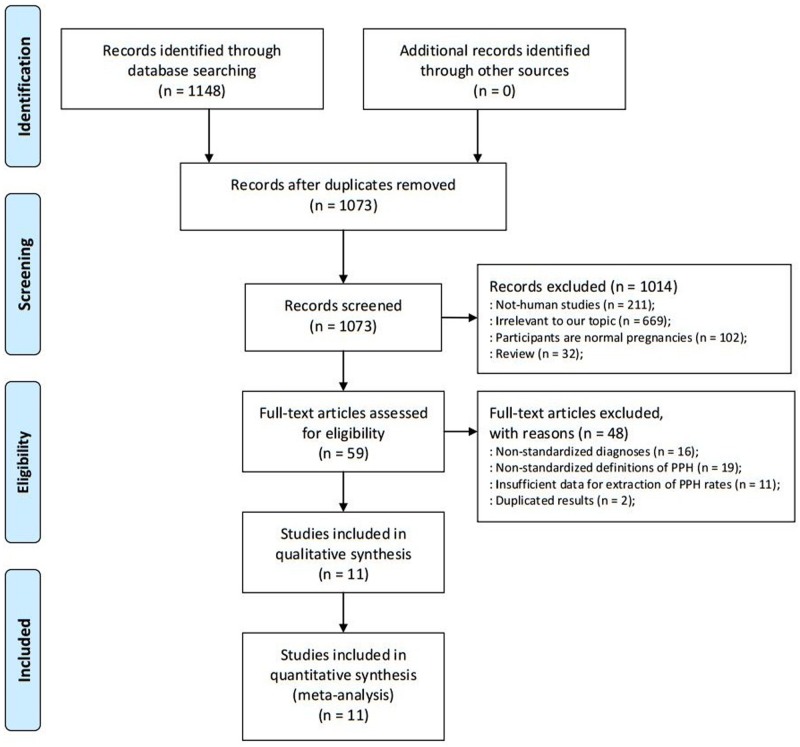
PRISMA flowchart showing the study selection process.

Our search yielded 1148 publications of which 11 studies including 14 unique PPH prevalence rates, met inclusion criteria (Fig 1). Five took place in North America [28–32], three in Asia [6, 33, 34], two in Europe [35, 36], and one in Australia [37] (Table 1). The articles were published between 2000 and 2016, and the final sample comprised 5146 unique pregnant women with placenta previa. Sample sizes ranged from 95 to 1612 participants with a mean sample size of 488. Mean age was 31.89 years (range = 29.78–34.20 years), and mean gestation age was 37.77 weeks (35.40–39.40). Five studies [28, 29, 34–36] reported anterior placenta frequencies and 36.05% of women (n = 860) were anterior placenta. 29.67% of women were prior cesarean delivery (n = 954), 67.73% of women were multiparae (n = 3322) and 6.85% of women were accompanied with placenta accrate. Six studies [28, 29, 31, 33–35] (n = 1329) including women were diagnosed with low-lying placenta previa. The prevalence of PPH ranged from 3.6% [31] to 58.7% [30]. When evaluated by STROBE quality assessment criteria, two studies [33, 37] received 9 points, five [29–31, 34, 35] received 8 points, and four [6, 28, 32, 36] received 7 points (S2 Table). The quality scores showed that studies were acceptable quality.

**Table 1:** Characteristic of included studies in meta-analysis.

Source	Cases	Total	Age* (years)	GA* (weeks)	AP	PCS	PA	MP	Somking	Survey Period	P (95% CI)	country	QS
Zhao L, 2016 [33]	54	312	29.85±2.62	32.62±3.76	---	30	17	211	---	2012–2015	0.17[0.13–0.22]	China	9
Wortman AC, 2016 [28]	42	98	30.75±6.10	38.07±2.30	18	21	---	80	3	2002–2012	0.43[0.33–0.53]	USA	7
Ji XL, 2015 [34]	43	112	32.00±5.43	---	38	34	27	80	---	2010–2014	0.38[0.29–0.47]	China	8
Osmundson SS, 2013 [29]	36	353	33.10±5.20	39.20±1.80	143	---	---	186	---	2009–2010	0.10[0.07–0.13]	USA	8
Ge J, 2012 [6]	213	1121	33.40±7.80	---	---	---	---	---	---	2005–2010	0.19[0.17–0.21]	China	7
Vergani P, 2009 [35]	20	95	34.18±5.60	36.78±4.30	46	---	---	36	---	2003–2008	0.21[0.13–0.29]	Italy	8
Zlatnik MG, 2007 [30]	135	230	162 (70.4%) < 35 years; 68 (29.6%) ≥ 35 years	35.40±2.50	---	180	---	135	---	1980–2001	0.59[0.52–0.65]	USA	8
Tuzovic L, 2006 [36]	32	202	75 (37.1%) < 30 years; 127 (62.9%) > 30 years	119 (58.9%) > 37 weeks; 47 (23.3%): 34–37 weeks; 17 (8.4%): 32–34 weeks; 11 (5.5%): 30–32 weeks; 8 (4.0%) < 30 weeks	65	20	14	170	40	1992–2001	0.16[0.11–0.21]	Croatia	7
Olive EC, 2005 [37]	309	1612	14 (0.9%) < 20 years; 1030 (63.9%):20–34 years; 568 (35.2%) ≥ 35 years	937 (58.1%) ≥ 37 weeks; 561 (34.8%):32–36 weeks; 93 (5.8%):28–31 weeks; 21 (1.3%):26–27 weeks	---	---	---	1118	---	1998–2002	0.19[0.17–0.21]	Australia	9
Ogueh O, 2003 [31]	25	703	31.30±4.80	39.40±1.80	---	82	---	---	76	1997–1999	0.04[0.02–0.05]	Canada	8
Crane JM, 2000 [32]	56	308	30	---	---	69	6	234	106	1988–1995	0.18[0.14–0.22]	Canada	7

AP: placenta on anterior wall; GA: gestational age; MP: multiperous; PA: placenta accrate; PCS: previous cesarean section; QS: quality score;

* Values indicate the mean (range), or mean ± standard deviation;

### Meta-analysis results

**Fig 2:**
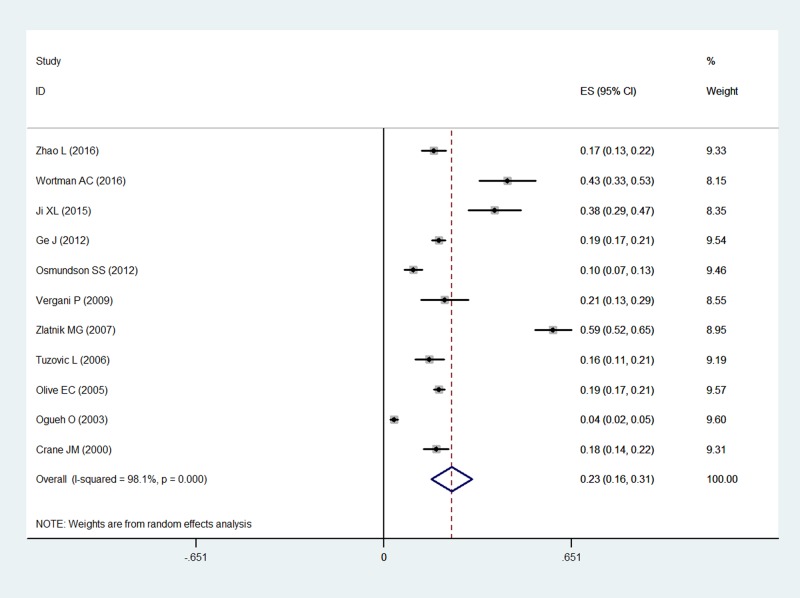
Forest plot of pooled estimated incidence of PPH with 95% CI.

**Fig 3:**
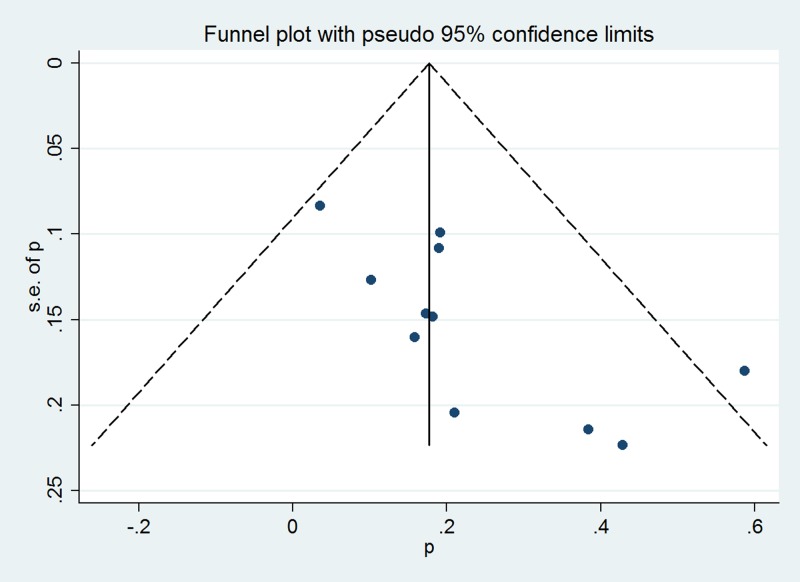
Funnel plot of the 11 studies included in the meta-analysis.

**Fig 4:**
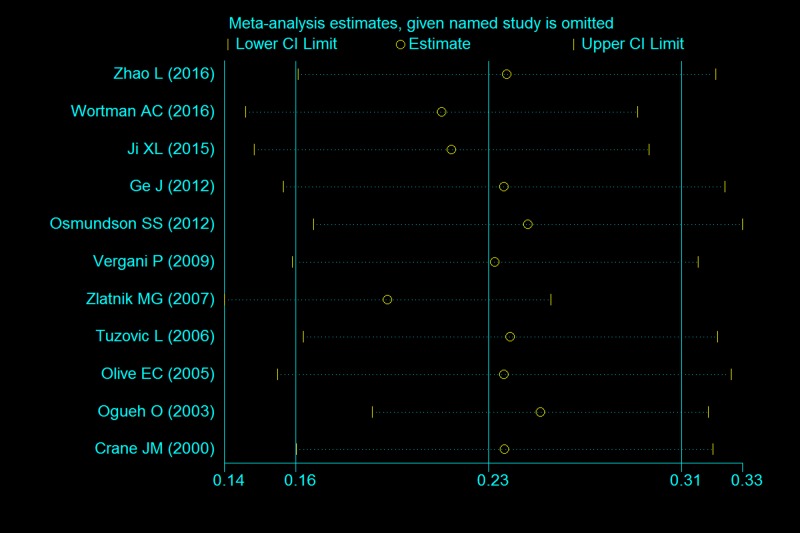
Sensitivity analysis for individual studies on the summary effect.

The overall pooled incidence was 22.3% (95% CI 15.8–28.7%). The *I*^*2*^ statistic (97.6%, P < 0.001) indicated substantial heterogeneity (Fig 2). The graphical funnel plots appeared to be symmetrical (Fig 3), and the Begg (z = 0.18, P = 0.855) and Egger test (t = -1.18, P = 0.263) indicated there was no strong evidence for publication bias. To confirm the stability and liability of the meta-analysis, sensitivity analysis was performed by repeating the calculation pooled PPH incidence when any single study was deleted. Fig 4 showed that the corresponding pooled incidence ranged from 19.7% (13.7–25.8%) to 25.4% (19.2–31.6%) and was not substantially altered. The statistically similar resulted indicated that each single study didn’t influence the stability of overall PPH incidence estimate in this meta-analysis.

### Subgroup results

**Fig 5:**
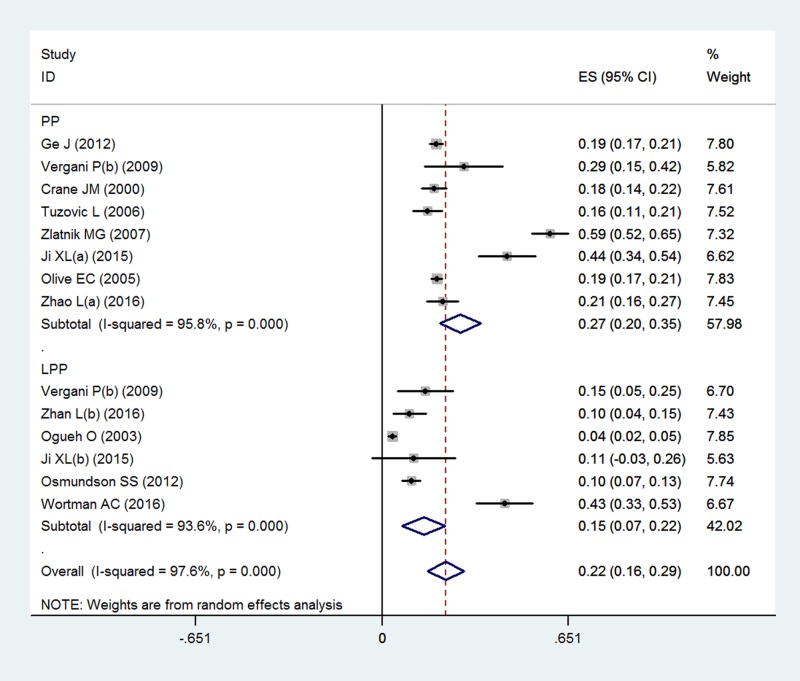
Funnel plot by subgroup analysis of placenta types. PP: placenta previas; LPP: low-lying placenta previa.

**Fig 6:**
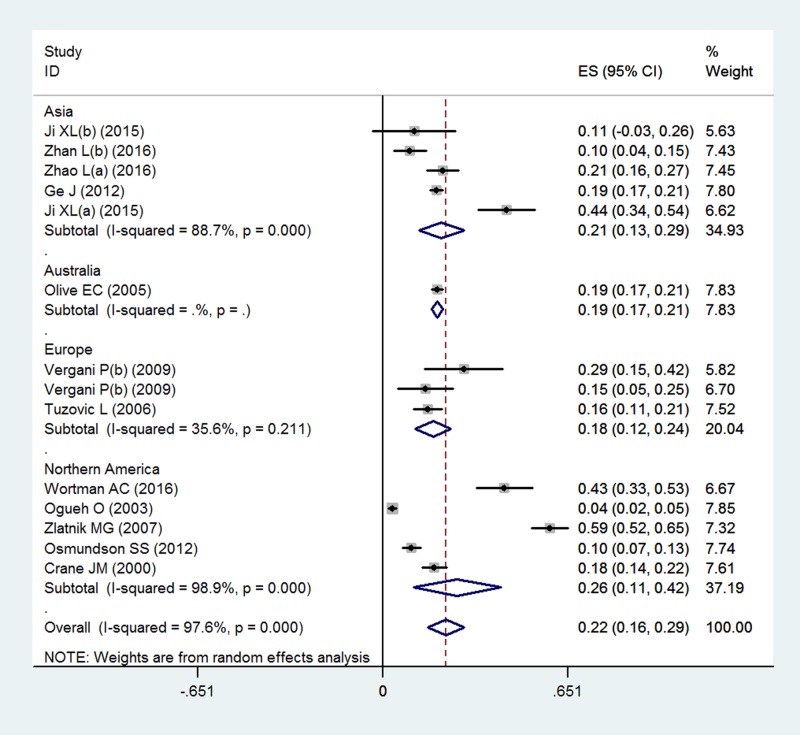
Funnel plot by subgroup analysis of world regions.

The PPH incidence was further analyzed by subgroup according to different types of placenta previa and world regions. The PPH incidence was 27.4% (95%CI 20.2–34.5%, n = 3817, *I*^*2*^ = 95.8%) in placenta previas, and was 14.5% (95%CI 7.0–22.1%, n = 1329, *I*^*2*^ = 93.6%) in low-lying placenta previa (Table 2 and Fig 5). The PPH incidence was lower in low-lying placenta previa (OR = 0.36, 95%CI 0.30–0.44, P = 0.001). Regarding the potential variations among world regions, the highest PPH incidence was estimated in Northern America (26.3%, 95%CI 11.0–41.6%, *I*^*2*^ = 98.9%), followed by the Asia (20.7%, 95%CI 12.8–28.6%, *I*^*2*^ = 88.7%), Australia (19.2%, 95% CI 17.2–21.1%, based on a single study) and Europe (17.8%, 95% CI, 11.5%-24.0%, *I*^*2*^ = 35.6) (Table 2 and Fig 6). However, it was found no difference in PPH incidence among the world regions (P = 0.227).

**Table 2:** Results of subgroup analysis for the incidence of postpartum hemorrhage.

Variable	Number of surveys	Total	Cases	P (95% CI)	*I*^*2*^ (%)
Placenta types					
PP	8	3817	842	0.27[0.20–0.35]	95.8
LPP	6	1329	123	0.15[0.07–0.22]	93.6
Regions					
Asia	5	1545	310	0.21[0.13–0.29]	88.7
Australia	1	1612	309	0.19[0.17–0.21]	---
Europe	3	297	52	0.18[0.12–0.24]	35.6
Northern America	5	1692	294	0.26[0.11–0.42]	98.9

PP: placenta previa; LPP: low-lying placenta previa;

### Meta-regression

A high level of heterogeneity between studies and subgroups was observed. Meta-regression was performed to explore potential sources of heterogeneity. Maternal age, gestational week, year of data collection, quality score, and percentage of anterior placenta (%), prior cesarean section (%), multiparous (%), placenta accreta (%), and smoking (%), which may be potential sources of heterogeneity, were tested by meta-regression method. Through the regression model, except for prior cesarean section (P = 0.044), none of aforementioned variables was significantly associated with the detected heterogeneity (Table 3). We, therefore, further tested the correlation between the percentage of prior cesarean section and the PPH incidence. It was found that there was a positive correlation between the percentage of prior cesarean section and the PPH incidence (r = 0.879, P = 0.049).

**Table 3:** Results of meta-regression for the incidence of postpartum hemorrhage.

Covariate	coefficient	95% CI	*t*-value	*P*-value
Age(year)	0.0060	-0.0415–0.0534	0.29	0.779
GA(week)	-0.0120	-0.0890–0.0570	-0.42	0.686
Survey year	-0.0040	-0.0163–0.0083	-0.71	0.490
QS	-0.0319	-0.1631–0.0994	-0.53	0.606
AP	-0.0103	-1.1385–1.1180	-0.04	0.972
PCS	0.6088	0.0285–1.1891	3.34	0.044
MP	-0.0768	-1.6342–1.4805	-0.13	0.904
PA	1.1018	-0.8807–3.0843	2.39	0.139
Smoking	-0.7846	-7.4064–5.8372	-1.51	0.373

AP: placenta on anterior wall; GA: gestational age; MP: multiperous; PA: placenta accrate; PCS: previous cesarean section; QS: quality score;

## Discussion

The aim of this systematic review and meta-analysis is to study the present status of the incidence of postpartum hemorrhage (PPH) in pregnant women with placenta previa (PP) and explore the determinants of PPH incidence. To our knowledge, this systematic review is the first meta-analysis and provides a comprehensive overview of the current literature. Based on the data of 11 articles including 14 unique studies, PPH incidence rate (22.3%) remains high, approximately 4-fold higher than among all women, in pregnant women with placenta previa. With reference to types of placenta previa, lower PPH incidence rate was demonstrated in low-lying placenta pregnant women (14.5%). When evaluated by study region, the incidence was high in North America (26.3%), intermediate in Asia (20.7%) and Australia (19.2%) and low in Europe (17.8%). In addition, it was also found that prior cesarean section associated with increased risk for PPH.

In 2008, a systematic review was published on the incidence of PPH with the objective of evaluating its magnitude both globally and in different regions [8]. Based on the results, the incidence was believed to be around 6% in observational studies. However, there was a wide variation across the different regions of the world, ranging from 2.55% in Asia to 10.45% in Africa. It was believed that risk factors for PPH included uterine atony, genital tract injuries, coagulation abnormalities, past history of PPH, multiple gestations and placental abnormalities, such as placenta previa or placenta accreta [13, 38, 39].

Several studies have estimated the incidence of PPH among pregnant women with placenta previa in different countries [30, 32, 33, 35]. However, there was a wide variation in the results of the conducted studies. The incidence of PPH was reported to be approximately 18% in a Canada population-based retrospective cohort study among 308 cases of placenta previa [32], 21% in a Italy retrospective singleton pregnancies cohort between January 2003 and August 2008 [35] and 59% in a USA retrospective singleton births cohort that occurred between 1976 and 2001 among the 230 placenta previa women [30].

Some studies have also focused on the association between placenta type and the risk of PPH. However, the results for this have been inconclusive. Ogueh et al [31] reported the incidence of PPH was only 3.56%, lower than among all women (6%), in a low-lying placenta women. Zlatnik et al [30] reported placenta previa was associated with PPH and the prevalence was even 10-fold higher (58.69%) for placenta previa than among all women. Risk factors of a wide variation include data collection period, sample size, placenta type, geographical region and other potential characteristics, such as prior cesarean sections, multiparous and gestational week.

Abnormal placentation has been observed to be associated with previous caesarean deliveries or other uterine surgeries, such as myomectomy or curettage, advanced maternal age and multiparity [4]. Previous studies have reported that these factors were associated with increased risk of PPH [13, 15, 38, 39]. In this study, our finding support existing evidence showing that prior cesarean section increases the risk of PPH by a system review and meta-analysis. However, similarly result was not found in other factors. The discrepancy could reflect a lack of statistical power in this system review owing to the small number of studies in other above factors.

The systematic review and meta-analysis included 11 articles including 14 unique studies with a large sample size, and it showed no potential risk of publication bias. The overall quality of the studies included was all acceptable, therefore, the result of sensitivity analysis was not substantially altered. Nevertheless, there are limitations due to the heterogeneity should be considered when interpreting the findings of this study.

The primary limitation of this meta-analysis was that significant heterogeneity between studies was observed in the study, which was not surprising as heterogeneity often exists in such meta-analysis of overall prevalence [40–42]. Although subgroup and meta-regression analyses did indicate that percentage of prior cesarean section to explain the observed heterogeneity, the remainder among the studies could be unexplained by the variable examined. Further analyses could not be performed, because of the limited information on these aspects. In addition, the results relied on aggregated published data. Further large-scale, multicenter prospective study using a single validated measured of PPH in a random subset of participants would provide a more accurate estimate of the incidence of PPH in women with placenta previa.

In conclusion, the summary estimate of the incidence of PPH among women with placenta previa was considerable in this systematic review. The results will be important for informing efforts to prevent, treat, and identify causes of PPH among pregnant women with placenta previa and would be contribute to the planning and implantation of relevant public health strategies.

## Supporting Information

S1 Checklist PRISMA Checklist. (DOC)

S1 Protocol Protocol. (DOC)

S1 Search Strategy The PubMed search strategy. (DOCX)

S1 Table The items of the chosen system for quality assessment of the included studies. (DOC)

S2 Table The score assignment to included studies. (DOC)
