# Supplementary material for: The Incidence of Postpartum Hemorrhage in Pregnant Women with Placenta Previa: A Systematic Review and Meta-Analysis
Source: PLoS One. 2017 Jan 20;12(1):e0170194. doi: 10.1371/journal.pone.0170194 (PMC5249070; doi:10.1371/journal.pone.0170194)
Supplement: S1 Protocol — (DOC) [file pone.0170194.s002.doc]

**S1 Protocol**

**Research protocol for systematic review**

**Title:** The incidence of postpartum hemorrhage in pregnant women with placenta previa: A Systematic Review and Meta-analysis.

**Authors:** Dazhi Fan^1,2¶^, Qing Xia^2¶^, Li Liu^2,3^, Shuzhen Wu^1^, Guo Tian^3^, Wen Wang^1^,

Song Wu^3^, Xiaoling Guo^1&^*, Zhengping Liu^1&^*

^1^ Department of Obstetrics, South Medical University Affiliated Maternal & Child Health Hospital of Foshan, Foshan, Guangdong, 528000, China

^2^ Department of Epidemiology and Biostatistics, School of Public Health, Anhui Medical University, Hefei, Anhui, 230032, China

^3^ Department of Library, the First Affiliated Hospital, College of Medicine, Zhejiang University, Hangzhou, Zhejiang, 310003, China

^4^ School of Integrated Traditional and Western Medicine, Anhui University of Chinese Medicine, Hefei, Anhui, 230038, China

*Corresponding author

E-mail: [liuzphlk81@outlook.com](mailto:liuzphlk81@outlook.com) (ZL); [fsguoxl@163.com](mailto:fsguoxl@163.com) (XG)

^¶^Dazhi Fan and Qing Xia contributed equally to this work and should be considered co-first.

^&^These authors also contributed equally to this work.

Rationale:

The global burden of postpartum hemorrhage (PPH) in women with placenta previa is a major public health concern. Although there are different reports on the incidence of PPH in different countries, to date, no research has reviewed them. The aim of this study was to calculate the average point incidence of PPH in women with placenta previa.

Objectives:

Purpose and intended goals of the literatur review:

- This study is designed to systematically review the present relevant studies which reported the incidence of PPH in pregnant women with placenta previa and has a pooled analysis of the prevalence in the overall population and subgroups of the participants.
- An attempt is also made to identify risk factors for the incidence of PPH by applying meta-regression analyses to the available data.

We will use PICOS criteria to improve the explicitness of the review questions:

Participants: Pregnant women with placenta previa.

Intervention/exposure: Pregnant women with PPH.

Comparisons: Postpartum hemorrhage in pregnant women with placenta previa

Outcome: The incidence of postpartum hemorrhage in pregnant women with placenta previa.

Study design: Observational studies (cross-sectional, retrospective and prospective studies).

**Methods**

Protocol and registration:

Our protocol and systematic review is conducted according to the PRISMA statement[1].

Eligibility criteria:

Inclusion

- Placenta previa was defined as a placenta that by ultrasound was partially or completely covering the internal os of the cervix;
- PPH diagnosis according to blood loss of more than 500 ml for vaginal deliveries and more than 1000 ml for cesarean delivery by the American College of Obstetricians and Gynecologists (ACOG);
- There were no language restrictions or time restrictions.

Exclusion

- Non-standardized diagnoses;
- Non-standardized definitions of PPH;
- Insufficient data for extraction of PPH rates;
- Case reports, letters, review articles or editorials;
- The full data was not accessible even after request from the primary/corresponding authors;
- Duplicated results;

Information sources:

Medline, Elsevier Science Direct, Cochrane Library and the Chinese National Knowledge Infrastructure database (CNKI). Relevant eligible literatures were also scanned through cross-references of identification in the reference lists within both original and review articles.

Search strategy:

Key words used were “placenta previa” OR “Low-lying placenta” OR “PP” AND “hemorrhage” OR “haemorrhage” OR “vaginal bleeding” AND “postpartum” in the title, abstract or index term fields.

Study selection:

Two independent authors (DF, LL) searched from database inception to 31 Jul 2016.

Data collection process:

Two reviewers (DF and SW) independently and carefully evaluated the articles and performed the data extraction according to the selection criteria. We extracted the following variables: first author, year of publication, survey years, study country, age (mean ± standard deviation or median, range), the number of cases of PPH and the total of placenta previa sample size. When discrepancies existed, discussion was performed via consultation with another reviewer (ZL) until a consensus was reached.

Quality assessment:

The methodological quality of each study was independently assessed by two reviewers (DF and QX) via the Reporting of Observational Studies in Epidemiology (STROBE) guideline. The STROBE guideline, which was a checklist of 22 items, included 5 core components (sample population, sample size, participation rate, outcome assessment, and analytical methods to control for bias). Each core component has three options: low risk (score = 2), moderate risk (score = 1), and high risk (score = 0). The total score which ranged from 0 to 10, represented the summary assessment of bias risk for each study. When there was a disagreement, it was solved by consensus of the whole team.

Statistical analyses:

Individual and pooled incidence as well as 95% confidence interval (95%CI) were calculated for each of all the included studies using the STATA 12.0 (Stata-Corp, College Station, TX, USA). The inverse variance methods and DerSimonian-Laird random-effects model meta-analysis was used to determine the weight of each study. To investigate potential sources of heterogeneity, subgroup analyses and meta-regression were performed to find any possible sources using the following grouping variables: type of placenta previa, geographical region, maternal age, gestational week, data collection period, percentage of potential characteristics (prior cesarean sections, multiparous, and anterior position of placenta) and study quality.

**Results:**

Depending on results, consider:

- Study characteristics and quality assessment;
- Meta-analysis;
- Subgroup analysis;
- Meta-regression analysis;

**Discussion:**

Summary of evidence

Limitations

Conclusions including implications for future research

**References**

1. Moher D, Liberati A, Tetzlaff J, Altman DG, Group P. Preferred reporting items for systematic reviews and meta-analyses: the PRISMA statement. International journal of surgery. 2010;8(5):336-41. doi: 10.1016/j.ijsu.2010.02.007. PubMed PMID: 20171303.
