# Supplementary material for: The Incidence of Postpartum Hemorrhage in Pregnant Women with Placenta Previa: A Systematic Review and Meta-Analysis
Source: PLoS One. 2017 Jan 20;12(1):e0170194. doi: 10.1371/journal.pone.0170194 (PMC5249070; doi:10.1371/journal.pone.0170194)
Supplement: S1 Search Strategy — (DOCX) [file pone.0170194.s003.docx]

**S1 Search Strategy**

Search ((((("Placenta Previa"[Mesh]) OR placenta previa) OR Low-lying placenta) OR PP)) AND ((((postpartum) AND (((hemorrhage) OR haemorrhage) OR vaginal bleeding))) OR "Postpartum Hemorrhage"[Mesh]) Filters: Publication date to 2016/07/31

*number of hits: 922*
