# Supplementary material for: The Incidence of Postpartum Hemorrhage in Pregnant Women with Placenta Previa: A Systematic Review and Meta-Analysis
Source: PLoS One. 2017 Jan 20;12(1):e0170194. doi: 10.1371/journal.pone.0170194 (PMC5249070; doi:10.1371/journal.pone.0170194)
Supplement: S1 Table — (DOC) [file pone.0170194.s004.doc]

Supplementary Table 1.The items of the chosen system for quality assessment of the included studies

| Bias type | Low risk (score=2) | Moderate risk (score=1) | High risk (score=0) |
| --- | --- | --- | --- |
| Selection (sample population) | 1. Sample from general population not a select group;  2. Consecutive unselected population;  3. Rationale for case and control selection explained. | 1. Sample selected from large population but selection criteria not defined;  2. Sample selection ambiguous but may be representative;  3. Rationale for cases and controls not explained;  4. Eligibility criteria not explained;  5. Analysis to adjust for sampling strategy bias. | 1. Highly select population making it difficult to generalise finding;  2. Sample selection ambiguous and sample unlikely to be representative. |
| selection (sample size) | 1. sample size calculation performed and adequate. | 1. Sample size calculation performed and reasons for not meeting sample size given.  2. Sample size calculation not performed but all eligible persons studied; | 1. Sample size estimation unclear or only sub-sample studied. |
| Selection (participation rate) | 1. High response rate (>85%). | 1. Moderate response rate (70–85%). | 1. Low response rate (<70%). |
| Performance bias (outcome assessment) | 1. Diagnosis using consistent criteria and direct examination. | 1. Assessment from administrative database or register;  2. Assessment from hospital record or interviewer. | 1. Assessment from non-validated data or generic estimate from overall population. |
| Performance bias (analytical methods to control for bias) | 1. Analysis appropriate for type of sample (subgroup analysis/regression etc.). | 1. Analysis does not account for common adjustment. | 1. Data confusing. |
