# Supplementary material for: The Incidence of Postpartum Hemorrhage in Pregnant Women with Placenta Previa: A Systematic Review and Meta-Analysis
Source: PLoS One. 2017 Jan 20;12(1):e0170194. doi: 10.1371/journal.pone.0170194 (PMC5249070; doi:10.1371/journal.pone.0170194)
Supplement: S2 Table — (DOC) [file pone.0170194.s005.doc]

Supplementary Table 2. The score assignment to included studies

| **Source** | **Selection**  **(sample population)** | **Selection**  **(sample size)** | **Selection**  **(participation rate)** | **Performance bias**  **(outcome assessment)** | **Performance bias**  **(analytical methods to control for bias)** | **QS** |
| --- | --- | --- | --- | --- | --- | --- |
| Zhao L, 2016 [33] | 2 | 1 | 2 | 2 | 2 | 9 |
| Wortman AC, 2015 [28] | 2 | 1 | 2 | 1 | 1 | 7 |
| Ji XL, 2015 [34] | 2 | 2 | 2 | 1 | 1 | 8 |
| Osmundson SS, 2012 [29] | 2 | 1 | 2 | 2 | 1 | 8 |
| Ge J, 2012 [6] | 2 | 1 | 2 | 1 | 1 | 7 |
| Vergani P, 2009 [35] | 2 | 2 | 2 | 1 | 1 | 8 |
| Zlatnik MG, 2007 [30] | 2 | 1 | 2 | 2 | 1 | 8 |
| Tuzovic L, 2006 [36] | 2 | 1 | 2 | 1 | 1 | 7 |
| Olive EC, 2005 [37] | 2 | 1 | 2 | 2 | 2 | 9 |
| Ogueh O, 2003 [31] | 2 | 1 | 2 | 2 | 1 | 8 |
| Crane JM, 2000 [32] | 2 | 1 | 2 | 1 | 1 | 7 |
